# Gravity in the brain—how it may regulate skeletal muscle metabolism by balancing compressive ischemic changes in the weight‐bearing pituitary and hypothalamus

**DOI:** 10.14814/phy2.14878

**Published:** 2021-05-27

**Authors:** J. Howard Jaster

**Affiliations:** ^1^ Department of Medicine London Corporation London UK

Recently in *the Journal*, a group of metabolism physiology investigators (Lamon et al., 2021) reported on their controlled study of sleep deprivation in normal healthy adults by looking at skeletal muscle protein synthesis, and specific hormone levels, as well as gene expression. They found that muscle protein synthesis was significantly reduced in sleep deprivation, compared to their control group. Sleep deprivation was also associated with an increase in plasma cortisol levels, and a decrease in plasma testosterone levels, compared to controls. Analysis of gene expression via muscle biopsy revealed no significant difference between sleep deprivation and the control group, suggesting no alteration of gene regulation during sleep deprivation.

A small collection of concurrent reports may potentially contribute to the “mechanistic insight’ sought by the metabolism physiology investigators—as to why sleep deprivation is associated with changes in muscle metabolism. A group of Norwegian sleep investigators (Voldsbekk et al., 2021) recently reported on a cohort of healthy volunteers in whom they studied sleep deprivation using novel brain magnetic resonance imaging (MRI) techniques to examine the white matter microstructure compared to a control group. They found significant white matter changes during sleep deprivation compared to during a normal sleep/wake cycle—which they considered to represent changes in axonal diffusivity.

And vascular laboratory investigators (Cherubini et al., 2021) elsewhere, studying endothelial function, may also have something to add. They have spent the last decade establishing an association between sleep deprivation and endothelial dysfunction. This endothelial dysfunction would potentially include the microvasculature of white matter in the brain, as well as the microvasculature of muscle. The works of these seemingly divergent groups of investigators might raise the question of whether vascular endothelial dysfunction plays a role in the brain white matter changes seen during sleep deprivation (Jaster, 2021) or in the metabolic changes seen in muscle (Lamon et al., 2021).

Part of the answer to this puzzle may be gravity—or more specifically, gravitational ischemia in the brain. Unlike the heart, lungs, and musculature, the brain sits on a hard floor of bone, where it is almost entirely motionless. The brain is analogous to a bed‐ridden hospital patient, who can avoid ischemic skin breakdown and decubitus ulcer formation only by the relentless efforts of a skin nurse to intermittently roll him from side to side, and reposition his arms and legs, to relieve under‐lying skin pressure resulting from the weight of over‐lying body mass in the earth's gravitational field (Jaster, 2021).

How does the brain do it? Does the brain roll itself from side to side? Maybe so—by sleeping 8 h/day, which encourages a horizontal positioning of head and body—and by maintaining wakefulness for 16 h/day, which encourages a vertical positioning of head and body (Jaster, 2021, 2018, 2020, 2015; Jaster et al., 2019).

“Gravitational ischemia in the brain,” results from the mass effect of one part of the brain upon another in a gravitational field. In any given head position, the “top” half of the brain (farthest from the center of the earth) is sitting on the “bottom” half as a weight‐burden. Pancaking layers of progressively increasing weight from the over‐lying brain tissue compress blood vessels and reduce blood flow in the bottom layers, resulting in regional gravitational ischemia on the bottom side of the brain (Jaster, 2021).

The Norwegian investigators may have visually chronicled the slowly accumulating diurnal effects of gravity in the brain—its fluid shifts possibly related to the gradually worsening ischemia of weight‐bearing strata of neuronal axons in tracts, situated “underneath” the mass of higher strata of brain tissue. Ischemia has long been an important pathological process in the heart, related to vascular stenosis, occlusion, and other phenomena—and so has vascular endothelial dysfunction. But observations about gravity in the brain are challenging the role of these two processes there, and whether each process may have two separate roles in the brain—a physiological role in metabolism, and a pathological role in stroke.

The skeletal musculature collectively is the body's anti‐gravity organ. It lifts both itself and the remainder of the body as it performs purposeful movements as directed by the nervous system. Its metabolism (and resulting size and strength) may be regulated directly by cortisol and testosterone levels—but it may be regulated indirectly by cortisol and testosterone releasing factors in the brain, active along the hypothalamic–pituitary axis. And this may be influenced significantly by gravitational ischemia in the brain (Jaster, 2021b, 2021; 2020).

The pituitary gland sits on a hard floor of sphenoid bone, within the sella turcica, giving it a certain specific susceptibility to gravitational pressure and ischemia, underneath a large mass of brain tissue, when the head is in vertical upright position. The rounded shape of the sellar floor provides gravitational resistance from several different directions (head tilting positions). In contrast, the hypothalamus is surrounded by soft brain tissue.

It appears at this time that explanations for phenomena such as “cortisol awakening burst” may not be low hanging fruit for physiology investigators, but may rather require elaborate computer modeling and mapping of changes in gravitational ischemia in different parts of the brain over time. At this moment, we are very far from understanding how these processes work, yet it is interesting to consider that regulation of the body's anti‐gravity organ may be accomplished in part by—well, … gravity.

In comparison, it may be interesting to look at two recent retrospective studies of patients who were probably "positionally compromised,” but not intentionally sleep deprived. One study (Fiest et al., 2021) sought an etiology for the high mortality rate in critically ill patients who experience delirium—and who may spend upwards of 24 h/day in supine position, often mechanically ventilated, with the occipital (visual association) cortex and brainstem vital centers carrying significant gravitational burden. The other study (Ebadi et al., 2021) sought an etiology for chronic fatigue in COPD patients—who may spend upwards of 24 h/day in a “head elevated position” due to breathing difficulty, and with both the hypothalamic‐pituitary axis and reticular activating system carrying significant gravitational burden. Neither study mentioned gravitational ischemia in the brain—and nor the study found the explanation it sought.
